# The Potential of Wire Explosion in Nanoparticle Production in Terms of Reproducibility

**DOI:** 10.3390/ma17143450

**Published:** 2024-07-12

**Authors:** László Égerházi, Tamás Szörényi

**Affiliations:** 1 Department of Medical Physics and Informatics, Albert Szent-Györgyi Medical School, University of Szeged, H-6720 Szeged, Hungary; egerhazi.laszlo@med.u-szeged.hu; 2 Department of Optics and Quantum Electronics, University of Szeged, H-6720 Szeged, Hungary

**Keywords:** nanoparticles, copper wire explosion, reproducibility, optical extinction spectroscopy, particle size distribution, oxidation states

## Abstract

Aquasols produced by exploding copper wires represent complex systems in which identifying individual colloidal components poses challenges due to broad and multimodal size distributions and varying shares among oxidation states. To evaluate the reproducibility of copper wire explosion, the size distribution of metallic and oxidized colloidal components within the 10–300 nm diameter range was assessed. Classification of each individual particle into bins according to size and chemical composition was accomplished by reconstructing the recorded optical extinction spectra of three sols produced under identical conditions as the weighted sum of the extinction spectra of individual copper and copper-oxide particles, computed using Mie theory. Our spectrophotometry-based component analysis revealed differences in particle number concentrations of the mainly oxidized nanoparticles, corresponding to deviations observed in the ultraviolet portion of the extinction spectra. Notable uniformity was observed, however, in the number of metallic fine particles, consistent with agreement in spectral features in the visible range. Regarding mass concentration, practically no differences were observed among the three samples, with nano-to-fine ratios of copper particles agreeing within 0.45%. Despite the complex processes during explosion leading to limited reproducibility in the ratio of different copper oxidation states, very good reproducibility (54.2 ± 0.7%) was found when comparing the total copper content of the samples to the mass of the exploded copper wire.

## 1. Introduction

Wire explosion (WE), wherein a metallic wire undergoes explosive fragmentation upon exposure to a short high-current pulse [1,2,3,4], presents itself as an environmentally friendly and straightforward top-down physical method for nanoparticle synthesis [5,6].

Current pulses, with current densities ranging from 10^4^ to 10^6^ A/mm^2^ and durations spanning from nanoseconds to microseconds, generated upon the discharge of a capacitor through a wire, induce an explosive disintegration phenomenon, leading to the ejection of wire material into the surrounding environment in the form of metal droplets and vapor. The size distribution of the particles, ranging from tens of micrometers to a few nanometers in diameter, is predominantly influenced by the energy injected into the wire [7].

Underwater wire explosion offers significant advantages over wire explosion in vacuum or gaseous environments. The high-threshold electric field (>200 kV/cm) in water prevents plasma generation along the wire surface, which in gaseous environments results in energy loss. In water, the entire electrical energy is thereby efficiently injected into the wire for disintegration. Another advantage is the nearly incompressible nature of water, which allows for gradual expansion. While the high-threshold electric field facilitates achieving current densities exceeding 10^5^ A/mm^2^, the reduction in radial wire expansion velocity helps maintain high energy density throughout the entire explosion process. Additionally, this form of WE also simplifies product collection in the form of colloidal solutions [8].

While the technique itself is not inherently complex, it presents significant challenges in characterizing the end products. The extreme conditions and competitive phase transitions during the explosion result in particles ranging from nanometers to tens of micrometers in diameter, exhibiting a multimodal distribution depending on the energy input into the wire. This variability significantly complicates experimental determination of the full particle size distribution, making the process both intricate and time-consuming [2,9,10,11,12,13,14]. Additionally, the wire material’s susceptibility to oxidation leads to chemically diverse systems with varying proportions of oxidation states depending on particle size. 

The general features of WE products, as outlined above, are well documented in the literature. Peng et al. [2] produced nanoparticles by exploding slightly longer copper wires (40 mm and 50 mm) with similar radii of 74 µm, discharging a 10 µF capacitor charged to 4, 5, and 5.5 kV. The particle size distribution they derived from transmission electron microscopy extended up to 180 nm, peaking in the 10–40 nm range. X-ray diffraction analysis indicated the presence of predominantly copper and a small amount of cuprous oxide. Approaching the problem of size distribution from the perspective of the nucleation mechanism, Tkachenko’s group stated that, depending heavily on the energy injected in the wire, the size distribution of the metal particles could range from hundreds of micrometers down to nanometers [9]. They noted that the size distribution should be at least bimodal due to different particle formation mechanisms. Cho et al. compared the size distribution of particles produced in air and water by exploding silver wires of 0.3 mm diameter and 40 mm length using the energy stored in a 10 µF capacitor charged to 12 kV [10]. Figure 4 in their paper shows that the diameter of the particles spanned from 20 nm to 200 nm in water and from 50 nm to 3 µm in air. The size distribution of particles produced in air exhibited two distinct peaks near 200 nm and 2 µm, indeed. A common feature of all reports is that, for energies high enough to produce nanoparticles, the particle sizes range from a few nanometers up to micrometers [2,9,10,11,13], with a bimodal distribution [9,10,12,13] peaking around 20–40 nm for the nanoparticles [9,14].

The abovementioned complexity of the size distribution and chemical composition of WE products imposes rigorous demands on characterization methodologies. Although transmission electron microscopy and scanning electron microscopy provide detailed size and chemical information, and X-ray diffraction (XRD) reveals crystal size and average bulk composition, none of these techniques can conveniently address the multidimensionality of such aquasols. For coinage metal (nano)particles, optical extinction spectroscopy (OES) has emerged as a widely utilized approach for obtaining information concerning particle size, morphology, concentration, and process kinetics on an ensemble of particles as a whole [15,16,17,18,19,20,21]. Monitoring alterations in the chemical state during redox processes poses an additional analytical challenge. Nevertheless, OES presents a pragmatic and effective solution under such circumstances [1,19,22], even offering real-time insights [23,24]. When the size distribution of the probed particles is unimodal, with a narrow standard deviation, monitoring the surface plasmon resonance peak (SPR) proves sufficient [25,26,27]. However, for the broad and multimodal particle-size distributions and chemically diverse compositions characteristic of copper WE [2,9,10,28,29,30], changes in the spectral characteristics of the SPR band alone are no longer adequate.

Starinskiy et al. determined the size distribution of a gold aquasol by modeling it as a combination of two groups of gold particles with average diameters of 9 nm and 50–200 nm. They reconstructed the measured extinction spectrum by superimposing the extinction spectra for both populations, each derived from elementary extinction spectra calculated using Mie theory [31].

In contrast to this model, which has low degrees of freedom, we introduced a more advanced and flexible approach [32] that simultaneously classifies individual particles into bins based on chemical composition and size. A distinctive characteristic of this approach is its ability to provide particle-specific information on the oxidation state of individual particles, assigning a chemical composition to each particle size bin for chemically inhomogeneous ensembles, such as the products of copper WE. This technique, in which the extinction spectra of the sols were reconstructed as the weighted sum of the extinction spectra of individual copper and copper-oxide particles within the diameter range of 10–300 nm based on Mie theory, is facilitated by the fact that the extinction spectra of particles of different sizes and oxidation states exhibit unique features across the entire spectral domain of 200–900 nm in the UV-VIS spectrum. In addition to the expected gradual shift of the surface plasmon resonance (SPR) peak into the visible range, mainly associated with fine copper particles, a notable common trait observed in the extinction spectra of Cu, Cu_2_O, and CuO nanoparticles was their predominant extinction in the UV range, irrespective of their oxidation state. The calculated extinction spectra of copper nanoparticles exhibited distinct features in the ultraviolet (UV) region, showing a significant dependency on particle size and gradually transitioning into a secondary peak in the visible region with increasing particle size. However, the spectra of copper(I) and copper(II) oxide nanoparticles were characterized by a single sharp extinction peak in the deep UV, which red-shifted as the particle diameter increased. As fingerprint-like peculiarities, the spectra of CuO showed slight broadening towards longer wavelengths, while the descending portion of the Cu_2_O spectra appeared featureless.

The method outlined above was previously employed to assess the nanoparticle production efficiency of copper wire explosion [32] but is also suitable for investigating a sensitive, rarely addressed issue, namely the reproducibility of WE in term of end products, which may be of particular relevance due to the complex and unpredictable processes accompanying the technique. The significance of such a study is highlighted by the lack of literature explicitly addressing the reproducibility of WE, particularly in terms of the consistency of characteristics in products prepared under identical conditions. Interestingly, such results are not available in the literature either, where at least findings from repeated measurements are presented, based on which this issue could potentially be addressed indirectly. The relevance of posing this question is however evident, given that the processes involved in WE are highly non-deterministic, implying potentially weaker reproducibility of the technique compared to other established nanoparticle production methods. Through spectrophotometric analysis of three aquasols prepared under identical conditions by exploding copper wires at a voltage of 6 kV, we aimed to investigate the reproducibility level of copper WE, separating the issue into considering process stability regarding the size distribution and oxidation state of the resulting particle ensemble (both nanoparticles and fine particles), as well as the throughput of WE.

## 2. Materials and Methods

### 2.1. Experimental Details

**Figure 1:**
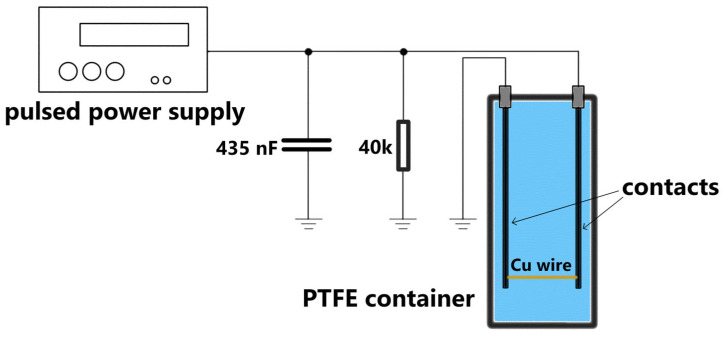
Experimental setup used for cupper aquasol generation by underwater wire explosion.

Three independent samples were prepared under identical conditions by exploding copper wires with a diameter of 70 µm and a length of 20 mm submerged in 100 mL of double-distilled water. The explosion was triggered by a high-current pulse in the microsecond range, generated by discharge of a 435 nF capacitor initially charged to 6 kV through a pulsed power supply (Figure 1). 

The aquasol to be analyzed was derived from the products of five consecutive explosions, with intervals typically lasting one minute. The particles were stabilized by continuous sonication in a commercial ultrasonic bath at room temperature (BANDELIN SONOREX RK102H, BANDELIN electronic GmbH & Co. KG, Berlin, Germany). This step was necessary since, after each explosion, the wire was manually secured between the contacts with a process duration sufficient to initiate primary nanoparticle aggregation. As documented by Lee et al. [33], sonication effectively mitigates aggregation of Cu nanoparticles produced by wire explosions. Gentle sonolysis maintains stable temperature and chemical conditions, ensuring consistency in the end products by minimizing variations due to post-explosion factors [34]. Immediately after the last explosion, the extinction spectra of aliquots of approximately 4 cm^3^ were recorded in the 200–900 nm spectral range employing a SHIMADZU UV-2101PC UV–VIS double-beam spectrophotometer (SHIMADZU DEUTSCHLAND GmbH, Duisburg, Germany) with water in the reference arm using HELLMA QS 1.000 cuvettes (Merck KGaA, Darmstadt, Germany). After sedimentation, the extinction spectrum of the supernatant, originating from extinction of ions in the solution, was also recorded [27,35] and later considered as a baseline for the calculations.

### 2.2. Computational Details

**Figure 2:**
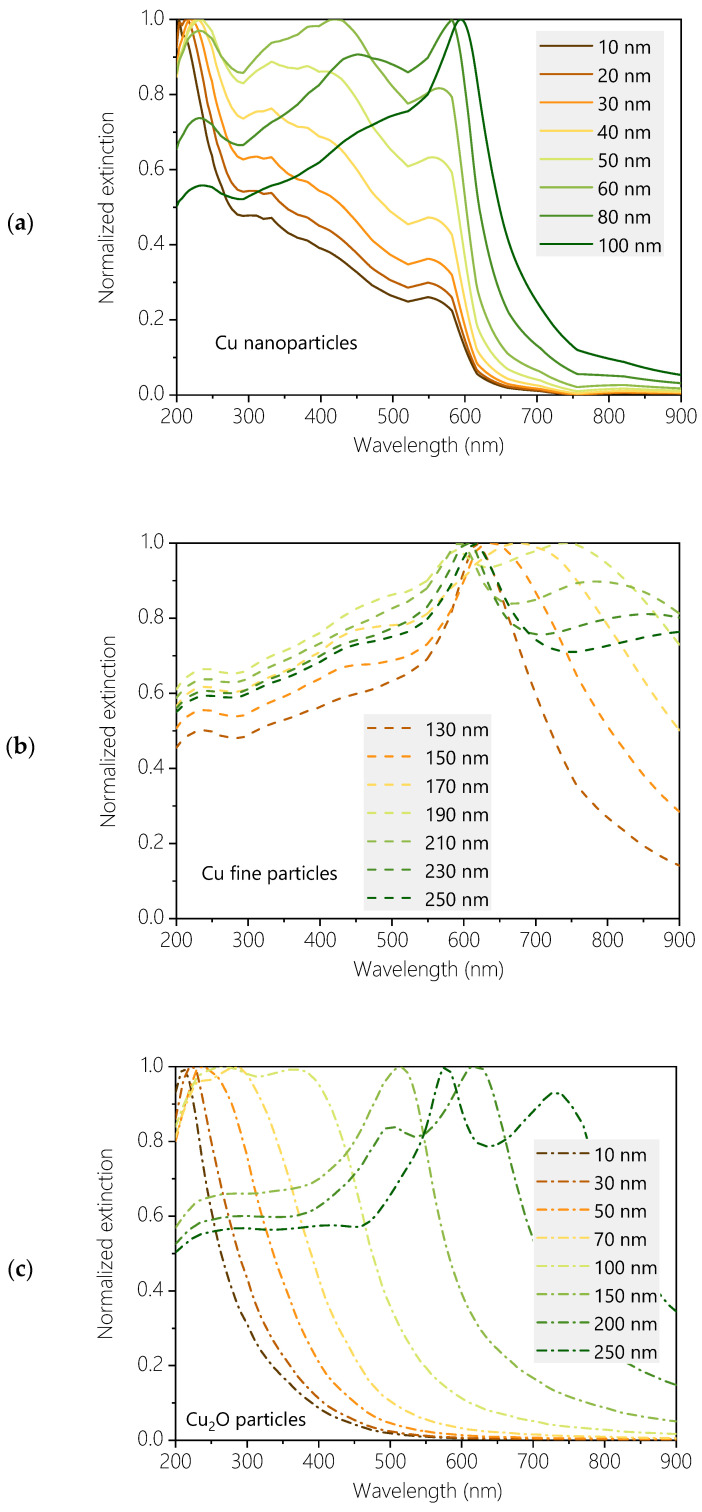

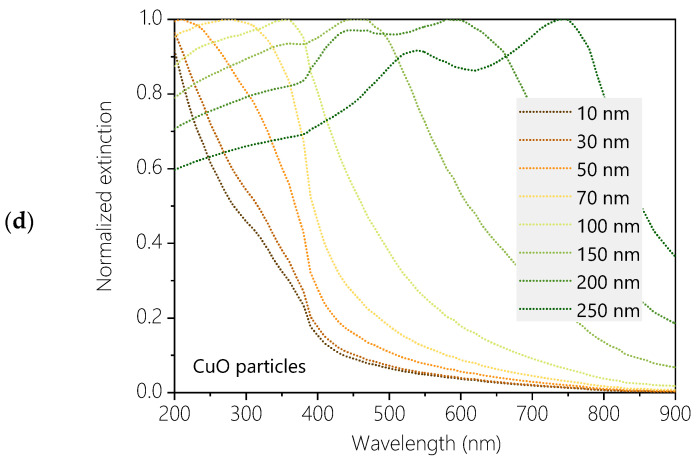
Computed extinction spectra of single Cu nanoparticles ((**a**) solid lines), Cu fine particles ((**b**) dashed lines), Cu_2_O particles ((**c**) dash-dotted lines), and CuO particles ((**d**) dotted lines) in water.

The size distributions and chemical compositions of the species in the aquasol were derived by approximating the measured extinction spectrum as the weighted superposition of the calculated extinction spectra of individual copper and copper-oxide particles with diameters ranging from 10 to 300 nm (in 10 nm increments) and the extinction spectrum of the supernatant. The extinction spectra of individual Cu, Cu_2_O, and CuO particles in an aqueous environment were computed using the classical Bohren–Huffman–Mie algorithm [36] with MiePlot v4.632 software by Philip Laven [37], assuming plane-wave illumination and a resolution of 0.5 nm in the 200–900 nm wavelength range, corresponding to the range and resolution of the measurements (Figure 2a–d). Numerical data were processed and plotted using Origin Pro 9.0 (OriginLab Corporation, Northampton, MA, USA).

The goodness of fit was characterized using the normalized root-mean-square deviation (NRMSD), defined as follows:

(1) $$NRMSD=\frac{1}{e_{avg,meas}}\sqrt{\frac{\sum_{i=1}^{n}{\left(e_{meas,i}-e_{mod,i}\right)}^{2}}{n}}$$

Here, *e_meas,i_* and *e_mod,i_* are the measured and modeled extinctions for the *i*-th wavelength, respectively, and *e_avg,meas_* and *n* represent the mean measured extinction and the number of data points along the investigated spectral range, respectively. The closer the model function is to the measured extinction curve, the lower the NRMSD parameter, ideally reaching zero in the case of a perfect match.

## 3. Results

**Figure 3:**
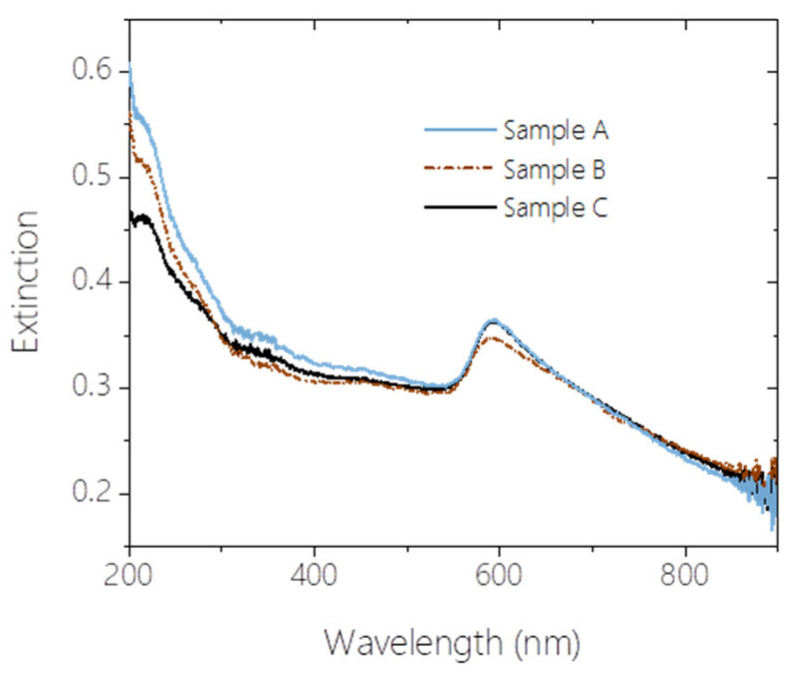
Measured extinction spectra of the three aquasols produced by copper WE at 6 kV.

Several common features in the shape of the recorded extinction spectra of the three aquasols, denoted as Sample A, Sample B, and Sample C in Figure 3, are apparent. From 200 nm to 300 nm, the spectral curves steeply decline, showing a distinctive shoulder at approximately 220 nm, which indicates the presence of Cu particles smaller than 70 nm in diameter. In the remaining UV region, a relatively broad shoulder around 350 nm suggests that the majority of copper nanoparticles are below 50 nm, as this shoulder typically shifts towards the 400–430 nm range for diameters above 50 nm. The enhanced UV extinction confirms the presence of Cu_2_O and CuO nanoparticles. These UV features collectively indicate the presence of nanoparticles [32]. Moving into the visible range, there is a gently declining plateau between 380 nm and 550 nm, followed by a pronounced, slightly asymmetric SPR peak with a maximum at 593 ± 1 nm, characteristic of fine copper particles, and a subsequent tail that declines almost linearly towards the infrared region.

Although the general appearances of the three extinction spectra are very similar, the individual extinction values for the three samples do not align across the entire investigated spectral domain. The most significant deviation among the three curves is observed in the deep UV region, reaching as high as +11.30%, +4.12%, and −15.38% for Samples A–C, respectively, at 200 nm, compared to the mean of the three extinction values. As the wavelength increases, this difference gradually diminishes, and the three functions converge. From 300 nm, the extinction of Sample C slightly exceeds that of Sample B, and this trend persists until 560 nm, where the three curves intersect. The SPR peaks of Samples A and C overlap with maximum extinctions of 0.3652 and 0.3636, respectively, whereas the maximum extinction of Sample B at the SPR peak is 4.3% lower.

**Figure 4:**
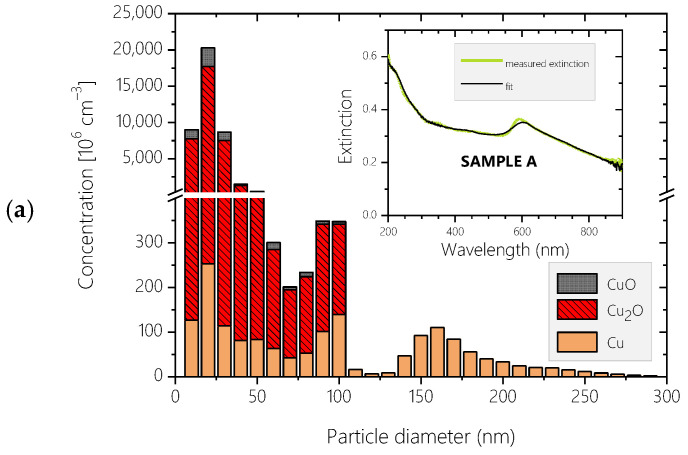

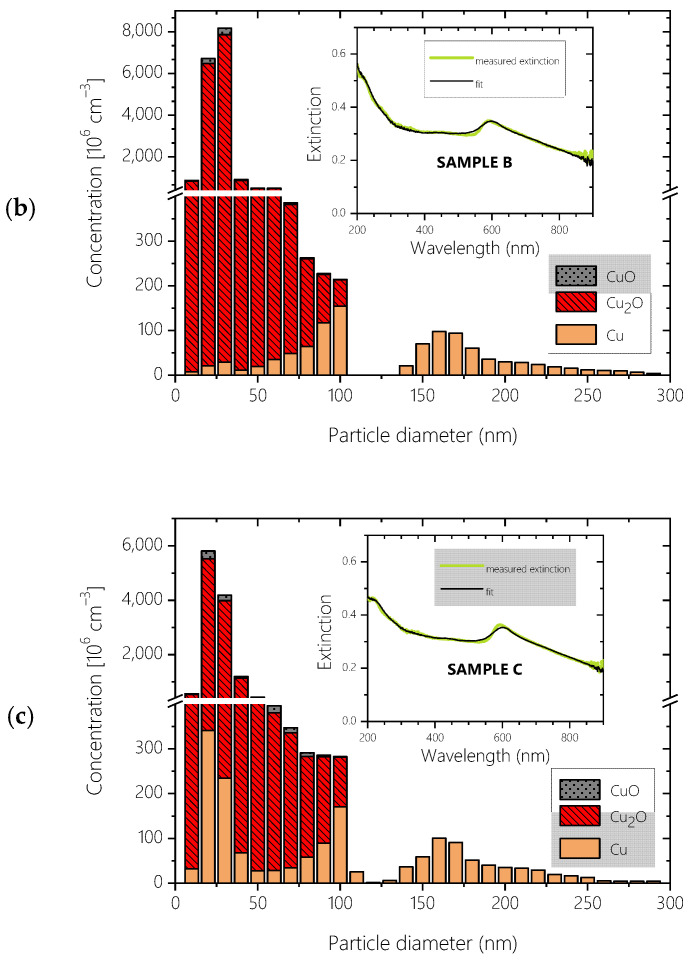
Particle size distributions and chemical compositions of Samples A (**a**), B (**b**), and C (**c**) produced by copper WE at 6 kV, derived from fitting the reconstructed spectra to the measured extinction spectra, as illustrated in the inset graphs.

Figure 4a–c depict the aggregated size distributions of the constituents of the three aquasols, represented by the particle number concentration of the considered metallic and oxidized components, as derived using the method outlined in the Computational details section. The inset graphs illustrate the recorded extinction spectra of the respective aquasols alongside the reconstructed spectra, which are the raw output of the modeling process. For each reconstructed spectrum, the NRMSD parameter, characterizing the goodness of fit, is below 1.40%. This low value serves as a prerequisite for obtaining reliable chemical and size information from the analysis.

In each distribution, nanoparticles (diameter ≤ 100 nm) and fine particles (diameter > 100 nm) particles form two distinct populations. The nanoparticles follow a log-normal distribution with maxima of approximately 20,300 × 10^6^ cm^−3^ (at a diameter of 20 nm), 8100 × 10^6^ cm^−3^ (at a diameter of 30 nm), and 5800 × 10^6^ cm^−3^ (at a diameter of 20 nm) for Samples A–C, respectively. Considering the percentage shares of the metallic and oxidized components in each size bin, for particles with diameters below 50 nm, the copper content slightly increases with particle size from 1% to 17%, 0.5% to 4%, and 5% to 6%, while the CuO content decreases concomitantly from 14% to 9%, 4.5% to 1%, and 5% to 4% for Samples A–C, respectively. The Cu_2_O content remains relatively independent of the particle size, amounting to 74%–85%, 95%, and 90%, respectively. For particles with diameters above 50 nm, the copper content increases significantly with particle size, reaching up to 40%, 72%, and 60% at a diameter of 100 nm. This increase is accompanied by a decrease in Cu_2_O content to 58%, 27%, and 39%, respectively, whereas the CuO content practically vanishes.

Compared to the nanoparticles, the fine particles are exclusively metallic, following a log-normal size distribution with less pronounced asymmetry and much flatter maxima of approximately 100 × 10^6^ cm^−3^ at 160 nm for all three samples.

## 4. Discussion

**Figure 5:**
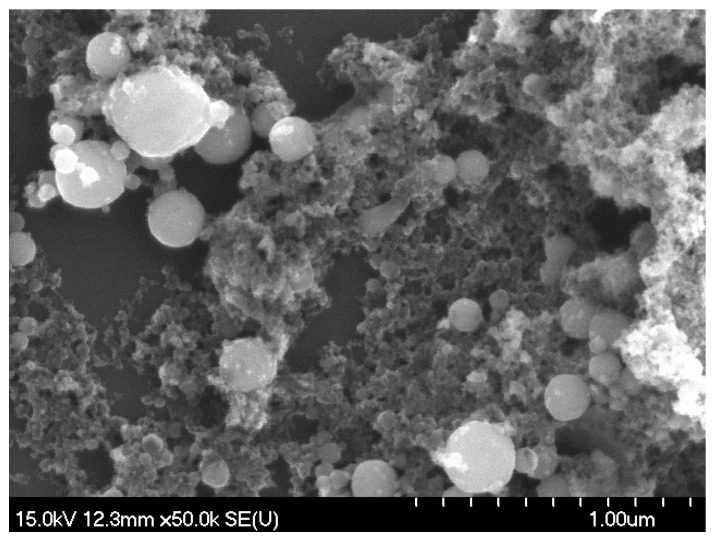
An SEM image taken of a 50 µL aliquot of Sample B dropped onto a carbon-coated grid and allowed to dry, showing the presence of two particle populations and confirming that there are significantly fewer fine particles (>100 nm in diameter) compared to nanoparticles.

Bimodal size distribution is a distinctive characteristic of wire explosion, independent of the wire material, resulting from different mechanisms operating in the two size domains [2,38,39,40]. Nanoparticles condense from the plasma phase, whereas fine particles are remnants of disintegrated liquid droplets [9,11,41,42]. The ratio of nanoparticles to fine particles in the distribution is influenced by the energy input into the wire. Oxygen ions in the plasma contribute to the formation of oxide nanoparticles, whereas the quasi-bulk fine particles, except for surface oxidation, retain the composition of the core. Experimental results also confirm the existence of two populations [43,44] and the log-normal size distribution of the nanoparticles [2,45,46,47,48] with a mode around 30 nm in diameter [28,32,44,46]. After exploding a copper wire of the same diameter at 8 kV, we obtained particle distributions consistent with the results presented in this article, with the sizes of fine particles ranging between 110 nm and 300 nm [32]. To further substantiate these characteristics, we present a SEM image (Figure 5) taken from a 50 µL aliquot of Sample B dropped onto a carbon-coated grid and allowed to dry. This micrograph, taken with a JEOL JSM-6480 LV scanning electron microscope (JEOL Ltd., Welwyn Garden City, UK) at 50,000× magnification, visually corroborates the presence of the two distinct particle populations and underscores the markedly lower number of fine particles in comparison to the nanoparticles.

The chemical compositions of the copper WE products have only been superficially addressed in a few studies [2,44,46,49]. Based on XRD studies, explosion products are usually reported to consist of copper with a small (<10%) oxide fraction [2,49,50], with Cu_2_O being the dominant oxidized form, even when WE is performed in a pure nitrogen atmosphere [2,44,46,50]. When preparing nanoparticles in deionized water and adjusting the energy injected into the wire over a broad range, the copper content decreased with increasing energy from 95% to 77% [44]. Occasionally, CuO was also observed in samples produced in air. Notably, no metallic copper nanoparticles were detected when a copper wire sample was exploded in air under atmospheric pressure [46].

**Figure 6:**
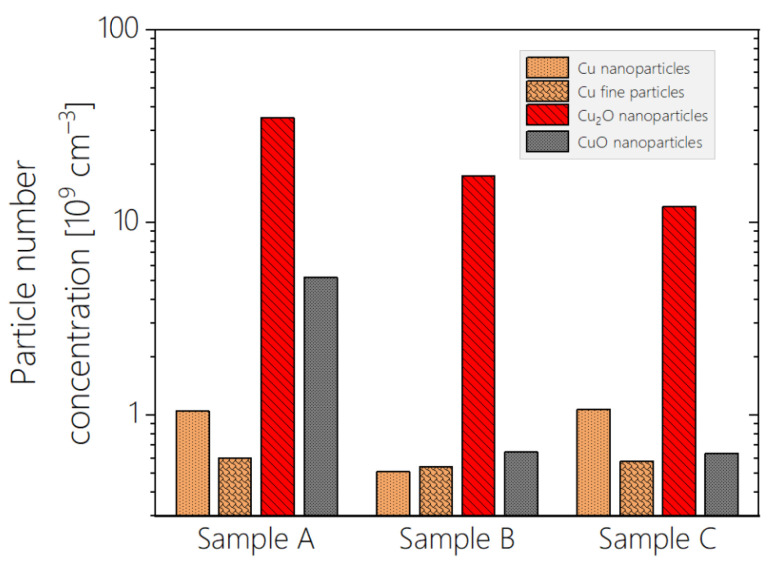
The aggregated particle number concentrations of copper fine particles and nanoparticles, as well as Cu_2_O and CuO nanoparticles, for Samples A–C produced by copper WE at 6 kV, as derived from the distributions in Figure 4.

Figure 6, an alternative projection of the distributions shown in Figure 4, presents the aggregated number concentrations of the copper fine particles and nanoparticles, as well as the Cu_2_O and CuO nanoparticles, for the three samples, using a logarithmic scale on the vertical axis. This comparative analysis also correlates the individual features of the three recorded extinction spectra with the peculiarities in the compositions of the samples.

As another representation, Table 1 illustrates the relative contributions of the constituents categorized by their size (nano or fine) and chemical state (metallic or oxide), both in terms of particle number concentrations and mass concentrations.

Figure 6 and Table 1 reveal that the majority of the particles in the sol are oxide nanoparticles (average particle number concentration: 93%), with Cu_2_O being the dominant species, which is consistent with observations in [2,44,46,50]. The average metallic content of the nanoparticles is less than 5%, and the fine particles are composed of pure metallic copper (approximately 3% of the total particle ensemble on average). Since extinction scales with particle number concentration, the high UV extinction of the sols is associated with the high relative concentration of copper-oxide nanoparticles, whereas the SPR peak around 590 nm is related to the copper content of the samples. The differences in number concentrations of the oxidized nanoparticles explain the varying UV extinctions of the three samples: lower concentrations of these species correspond to lower UV extinction, as shown in Figure 6 and the second row of Table 1. Conversely, the highest relative content of copper nanoparticles in Sample C is manifested by a pronounced extinction peak at 220 nm (see Figure 3), and this high copper-to-copper oxide ratio in the 20–30 nm particle size domain is responsible for the elevated extinction around 330 nm. 

The extinction spectra of the nanosized copper particles exhibit a cut-off wavelength at approximately 600 nm, contributing to the measured spectrum at wavelengths below this threshold. In contrast, the spectra of fine copper particles show pronounced extinction peaks in the 600–800 nm range, which are size-dependent. The total copper content of Samples A and C is identical within 1% (see Figure 6), explaining the similar behavior of their plasmon peaks. Sample B contains significantly less copper (63% compared to Samples A and C), resulting in less pronounced extinction around the plasmon peak. This deficiency is primarily in the nano region (the nano-to-fine copper particle ratios are 1.75, 0.94, and 1.86 for Samples A–C, respectively, as calculated from the first row of Table 1), leading to reduced extinction below 600 nm and a less elevated SPR peak tilted to the left. A deviation of no greater than 10% in the number concentration of fine copper particles among the three samples accounts for the lack of significant difference in the extinction spectra above 650 nm.

In the sections above, the particle number concentration statistics were discussed, since the raw data provided by OES reflect this aspect of the analysis. To compare our results with data from the literature obtained by XRD, the chemically selective size distributions of the particles were converted into a representation reflecting the mass concentrations (third and fourth rows of Table 1). The differences in mass concentrations among the three samples are less pronounced compared to the differences in particle number concentrations: the nano-to-fine ratios of the copper particles for the three samples are within 0.45%. The increased nanosized oxide content of Sample A in this representation is linked to the relatively high number concentration of nanoparticles in the >90 nm diameter domain. Furthermore, the largest contribution to the total mass of the samples originates from the fine copper particles. With 22.2 ± 0.1 g/m^3^, the total copper mass concentrations obtained from the metallic species and the copper content of the oxidized species in Samples A, B, and C are identical within 1%. This suggests that WE is highly reproducible in terms of throughput, defined as the ratio of the total copper mass in the sol to the copper mass exploded.

Since the mass of the species scales with the third power of the diameter, the contributions of oxidized particles (mostly nanosized) and metallic species (mostly fine) are inverted in the mass representation compared to the particle number representations shown in Figure 6. This relationship explains why several studies claim that the oxide content in copper WE products is relatively low [2,44,46,49,50], considering that XRD, used for characterization, is more sensitive to the bulk mass of the constituents.

## 5. Conclusions

Characterizing primarily the number concentration and chemical compositions of particles, the spectroscopy-based method presented herein can disclose minor differences in the end products of copper WE, which arise from the complex and unpredictable processes occurring during the explosion. All three oxidation states investigated (Cu, CuO, and Cu_2_O) were represented in the nanosized particle populations of the three samples produced in an aqueous environment at a charging voltage of 6 kV.

Our analysis, in agreement with the results derived from the analysis of sols produced at 8 kV [32], revealed that, in the particle concentration representation, the overwhelming majority of nanoparticles were oxidized. Cu_2_O was found to be dominant, and pronounced differences were observed in the ratio of the three oxidation states, explaining the differences in the UV part of the recorded extinction spectra. Additionally, high reproducibility was concluded for the fine particles, which proved to be exclusively metallic.

After converting the chemically selective size distributions presented in Figure 6 into mass concentrations (Table 1), it was evident that the metallic species were the major contributors to the total mass of the samples (86.7 ± 2.4%), consistent with XRD results available in the literature [2,44,46,49,50]. Despite the complex processes during the explosion, which result in poor reproducibility in the ratio of the different copper oxidation states, very good reproducibility can be identified when comparing the total copper content of the samples to the mass of the exploded copper wire (54.2 ± 0.7%).

Our findings affirm the weaker reproducibility of WE, expected due to the inherently unpredictable non-equilibrium processes, especially in terms of the nano-to-fine particle size ratio and the metallic-to-oxide chemical composition rate of the fine and nanoparticles. However, our results underscore that this variability is mainly reflected in the particle number concentrations, and, considering the mass concentration, the stability of the method is much more reliable. This study not only fills a gap in the literature regarding the reproducibility of WE but also highlights that spectrophotometry-based component analysis effectively translates qualitative differences observed in the extinction spectra of aquasols obtained from repeated experiments into quantitative distinctions in the chemical compositions and size distributions of the explosion products. Thus, serving as a complementary technique to other nanoparticle sizing methods (such as electron microscopy or atomic force microscopy), the particle system characterization technique we introduced offers significant potential due to its capacity to probe billions of particles. It ultimately enables determination of not only the size distribution of particles by measuring the extinction spectrum of the end product; in the case of a chemically heterogeneous ensemble, such as the products of copper WE, this method additionally provides particle-specific insights into oxidation state, assigning a chemical composition to each particle size bin, unlike XRD, which provides bulk information.

## Figures and Tables

**Table 1:** The relative particle number and mass concentration contributions of the constituents of Samples A–C, categorized by their sizes and chemical states.

		Sample A		Sample B		Sample C	
		Nanoparticles	Fine Particles	Nanoparticles	Fine Particles	Nanoparticles	Fine Particles
**Percent contribution** **(in number concentration)**	**metallic**	2.52%	1.44%	2.65%	2.82%	7.53%	4.04%
	**oxide**	96.03%	0.00%	94.53%	0.00%	88.43%	0.00%
**Percent contribution** **(in mass concentration)**	**metallic**	6.10%	77.92%	6.38%	81.64%	6.32%	81.09%
	**oxide**	15.98%	0.00%	11.98%	0.00%	11.82%	0.00%

## Data Availability

The raw data supporting the conclusions of this article will be made available by the authors on request.
